# Preferred Placement and Usability of a Smart Textile System vs. Inertial Measurement Units for Activity Monitoring

**DOI:** 10.3390/s18082501

**Published:** 2018-08-01

**Authors:** Mohammad Iman Mokhlespour Esfahani, Maury A. Nussbaum

**Affiliations:** 1 Department of Industrial and Systems Engineering, Virginia Tech, Blacksburg, VA 24061, USA; mokhlespour@vt.edu; 2 School of Biomedical Engineering and Sciences, Virginia Tech, Blacksburg, VA 24061, USA

**Keywords:** smart textile system, inertial measurement unit, wearable sensor, usability, placement, smart shirt, smart socks

## Abstract

Wearable sensors and systems have become increasingly popular in recent years. Two prominent wearable technologies for human activity monitoring are smart textile systems (STSs) and inertial measurement units (IMUs). Despite ongoing advances in both, the usability aspects of these devices require further investigation, especially to facilitate future use. In this study, 18 participants evaluate the preferred placement and usability of two STSs, along with a comparison to a commercial IMU system. These evaluations are completed after participants engaged in a range of activities (e.g., sitting, standing, walking, and running), during which they wear two representatives of smart textile systems: (1) a custom smart undershirt (SUS) and commercial smart socks; and (2) a commercial whole-body IMU system. We first analyze responses regarding the usability of the STS, and subsequently compared these results to those for the IMU system. Participants identify a short-sleeved shirt as their preferred activity monitor. In additional, the SUS in combination with the smart socks is rated superior to the IMU system in several aspects of usability. As reported herein, STSs show promise for future applications in human activity monitoring in terms of usability.

## 1. Introduction

Traditional tools and devices for conducting human movement studies are often cumbersome or uncomfortable for subjects. Recently, the development of wearable sensors has facilitated new measurement alternatives. These sensors are lightweight and portable, meaning that there is a potential for measurements to be obtained with minimal discomfort in diverse contexts, even in people’s daily life. Nonetheless, further innovations in wearable sensors are crucial to improved research and expanded applications. Over the past decade, two wearable systems have gained pre-eminence in this regard: microelectromechanical systems (MEMS) and smart textile systems (STSs) [1].

### 1.1. Microelectromechanical Systems (MEMS)

MEMS represent an advanced technology for manufacturing wearable systems for use in activity monitoring. One of the examples of a MEMS sensor for this purpose is accelerometers, which researchers have used in a wide range of applications. In the healthcare field, for example, it has been used to measure human activities during ambulatory applications [2,3,4,5], as well as for determining inclination during static and semi-static situations [6]. More generally, it has been used to develop activity monitoring systems for diverse applications, for example the ActiGraph and activePAL [7,8]. Specifically, an accelerometer is useful for measuring inclination when the body moves with moderate acceleration, compared to gravitational acceleration. While accelerometers are beneficial for assessing static and semi-dynamic motion, many human physical activities are dynamic, thus limiting the utility of accelerometers for postural assessment. There are a number of other limitations associated with accelerometry-based devices that should be noted: (a) low-sensitivity for sedentary behaviors [9], (b) the lack of standard or generally acceptable placements for them [10,11], and (c) limited ability for assessing low-intensity activities. 

Another MEMS device is the gyroscope, which measures the angular velocity of the body or body segment. Although it is useful for some applications, its signals tend to be accompanied by significant noise. Moreover, it has limited accuracy when the goal is to estimate angles via integration. To achieve more accurate kinematic measures for dynamic movements, the accelerometer and gyroscope have been combined into an inertial sensor [12].

A third common MEMS sensor for activity monitoring is the magnetometer, which was originally developed to measure the Earth’s magnetic field. Magnetometers have been combined with accelerometers and gyroscopes to increase the accuracy of orientation estimation in the vertical axis (yaw angle). This combination is known as an inertial measurement unit (IMU) [13,14]. In other words, an IMU consists of an accelerometer, a gyroscope, and an optional magnetometer. IMUs can be placed on body segments of interest to capture segmental kinematics (e.g., three-dimensional acceleration, angular velocity, and angular orientations). Over the last decade, several companies have produced whole-body measurement systems using IMUs, including Xsens (Xsens North America Inc., Culver City, CA, USA) and Biosyn (Bio-Synthesis Inc., Lewisville, TX, USA).

For accurate and drift-free measurements, several fusion algorithms have been developed, for example the Kalman filter and the more recent Attitude and Heading Reference System (AHRS); several studies [13,14,15,16,17,18] have used these algorithms to combine sensory outputs and thereby obtain accurate kinematic parameters during human movement. Table 1 provides an overview of different wearable sensors using MEMS technology.

In addition to current features of wearable sensors, there is one more important feature of a wearable system that should be considered. According to the level of integration into a garment, wearable systems can be considered as either an intrinsic type, in which sensing features are incorporated within the textile structure, or an extrinsic type, wherein the external sensing elements are attached to the fabric [27]. Most available devices for detecting and capturing movements, such as those mentioned above, can be considered as an extrinsic type. Despite the utility of external sensing elements for assessing physical activity (e.g., in daily life or in the workplace), these extrinsic systems have four notable drawbacks compared to the intrinsic type: (1) positioning a rigid object on a garment may decrease the wearer’s comfort and restrict their activities [28]; (2) the electronic components on the garment are not likely washable; (3) the power supply for these electronics may be limited, requiring the wearer to be vigilant about battery replacement or recharging; and (4) they may not be implemented comfortably in very close proximity to the skin, and thus may not be effective for measuring realistic behaviors.

### 1.2. Smart Textile Systems (STSs)

Given the potential limitations of existing systems (in terms of comfort, wearability, and validity), a recent and promising technology is emerging: the smart textile system (STS). In general, components of an STS include sensors, connectors, circuit boards, and energy sources [27,29], each of which is described below. A long-term goal is to integrate all required components of an STS into a garment, either intrinsically or extrinsically. With such integration, a fully integrated textile-based system is promising to be used for measuring diverse physiological and physical health-related factors both remotely and continuously [29].

**Sensor:** A smart textile sensor is a sensitive fiber, yarn, or fabric that transduces an external stimulus (e.g., physical or chemical) [27,29,30,31,32]. Pressure, temperature, heart rates, optical parameters, and muscle strain are several examples of features that smart textile sensors can measure [27,29,31,32,33,34,35,36].

**Circuit boards and connectors:** Circuit boards are needed to convert analog signals to digital outputs, while connectors transfer the textile sensor signals to circuit boards. Investigators have used diverse approaches for adding a connector or a circuit to a garment, such as snaps, embroidered conductive yarns, and embedded copper wires [27].

**Energy sources:** To augment the potential of smart textile systems, investigators are working to develop textile-based, energy-harvesting materials [37,38,39].

Table 2 lists a range of commercial textile sensors and their use in medical applications. Note that this table includes recent scholarly references, which will no doubt expand as researchers continue to develop the potential of textile sensor technology. 

### 1.3. Usability Perspective

As noted above, both IMU and STS might be useful in a number of applications, but these technologies can also be limited by certain environmental and data collection aspects. Thus, a number of researchers are designing wearable systems based on one or both technologies and implementing them in diverse situations [1]. Importantly, these researchers must take into account another critical factor when they design and implement a wearable system—aspects and parameters, which are important to the user to support the design of effective wearable systems that are compatible with real-world applications. Particularly, the usability of any given system is strongly associated with a user’s trust that the information provided by wearable devices is accurate and informative, which encourages the user to continue wearing the device [58]. Additional research, therefore, is needed to help determine the benefits and limitations of each available technology according to the perspective of users.

A number of studies have indeed investigated the usability of wearable sensors to support their implementation during diverse applications [59,60,61], which have principally involved MEMS technology as an external device. For example, in their study of a fall-monitoring system among elderly people with Alzheimer’s disease, Abbate et al. [62] evaluated seven usability criteria, such as willingness to use, ease of learning, levels of satisfaction, and interface with activities of daily living. Furthermore, Schall et al. [63] assessed the benefits of and barriers to using wearable sensors in the workplace, and showed that respondents were interested in using wearable sensors, but had concerns about the privacy of collected data. Beeler et al. [64] investigated the comfortability and acceptability of nine wearable devices for the use of soldiers, and found that placements of wearable sensors on the upper arm, hip, and shoes were preferred by this group [64]. Bergmann et al. [60] showed that patient-participants preferred to use a small and unobtrusive wearable system positioned at the upper extremity over an extended period of time. In their study of emergency room patients, Claudio et al. [65] investigated the usefulness and ease-of-use of a wearable device to monitor vital health parameters, such as heart rates, respiration rates, and blood pressures. In both of the latter studies, users indicated that activity-monitoring devices should be lightweight, compact, easy to carry, and simple to operate, as well as have a long battery life [59,65].

Despite the growing number of studies in this realm, no study, to the best of our knowledge, has explored the comparative usability of STSs and. IMUs. The aim of this study was thus to compare the usability of these two smart textile systems, both individually and in combination, with an IMU system. We also assessed preferred placements of these devices as a supplement to earlier reports.

## 2. Materials and Methods

### 2.1. Participants and Experimental Procedures

Eighteen participants (12 males and 6 females) completed the current study, which was conducted in a laboratory environment. There were two inclusion criteria: (a) participants had to be free of any self-reported current or recent musculoskeletal disorders (prior year); and (b) a smart undershirt used herein and described below had to fit participants properly (snug but not uncomfortable), since it was only available in a single size. Summary information about the participants is provided in Table 3. The study protocol was approved by the Institutional Review Board at Virginia Tech, and all participants provided written consent prior to participation.

**Figure 1:**
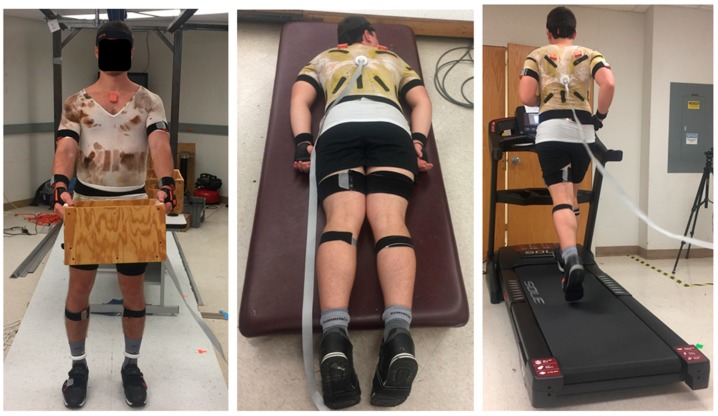
Illustrations of a participant wearing two smart textile systems (STSs) (an SUS and smart socks) and a full-body inertial measurement unit (IMU) system during a sample of activities: carrying a box (**left**), laying down (**middle**), and running on a treadmill (**right**).

Participants were asked to perform a number of specified activities, including sitting, standing, walking, running, stair climbing up/down, laying down, manual material handling tasks (e.g., lifting, carrying, pulling, and pushing the box), and simple upper body movements (e.g., shoulder flexion/extension, abduction/adduction, and internal/external rotations, as well as 3D rotations of lumbar spine). Each activity lasted for approximately 2 min. The complete set of activities was completed in approximately 3 h sessions, while participants wore three wearable activity monitoring systems. Two STSs (a smart undershirt (SUS) and smart socks) and a full-body IMU system were included (Figure 1), described in more detail subsequently. 

### 2.2. Wearable Systems

#### 2.2.1. Smart Textile Systems

Two distinct STSs were selected: an SUS and smart socks. We chose these two systems as examples of technology for assessing movements of the upper and lower extremities, respectively. The former was developed in our prior work [57], while the latter is a commercially-available product called Sensoria socks [51]. The SUS consists of 11 polymer-based textile sensors coated on an undershirt that measure fabric strains, and additional details have been reported elsewhere [53]. The SUS captures both low-back and shoulder motions, which at present has a hard-wired connection to a computer. (Note: an SUS is under development that can transfer data wirelessly.) Only a single-sized smart shirt was available, while Sensoria socks in several sizes (small, medium and large) were ready to use. All participants were asked to wear standard athletic shoes for the possible effects of different types of shoes. The Sensoria socks feature three textile pressure sensors in three specific placements on the bottom of the sock: at the heel, under the first metatarsal bone, and under the fifth metatarsal bone. Unlike the SUS, data from the smart socks could be transmitted wirelessly. 

#### 2.2.2. Inertial Measurement Unit System

A commercial, wireless IMU system (MTw Awinda, Xsens Co., Enschede, The Netherland) was used as an example system for capturing whole-body kinematics. This system includes 17 IMU sensors placed on the head, upper arms, lower arms, hands, scapula, chest, pelvis, upper legs, lower legs, and feet. The dimensions and the mass of each sensor are 47 × 30 × 13 mm and 16 g, respectively. Sensors were attached to the relevant body segments using a stretchable strap, except for those located on the scapula and chest attached with a surgical tape. Of note, the MTw Awinda comes with a full-body garment with “pockets” to hold the individual sensors. We did not use their garment for two reasons. First, it would have interfered with the SUS. Second, we presumed that most users of such a system, in practice, would wear a subset of IMUs, or even a single sensor, with more localized methods of attachment.

### 2.3. Assessing Usability

To evaluate the relative usability of the two systems, participants completed two-part questionnaires for each system. In the first part, they indicated their preferred placement for IMUs and preferred garments for an STS (as many as they wished to note). They were asked to picture themselves using these systems to monitor their daily activities, or to imagine a health professional using it for medical applications. In the second part of each questionnaire, they answered 14 questions for each system. Participants responded to each question using five-level Likert-type scales (e.g., 1 = strongly disagree, 5 = strongly agree). These questions addressed a range of usability aspects:

(1) Is it comfortable?
(2) Is it small enough?
(3) Is it lightweight?
(4) Is it safe?
(5) Is it simple to use?
(6) Is it fashionable?
(7) Does it motivate me to use it?
(8) Will it disturb my privacy?
(9) Does it interfere with the appearance of a garment?
(10) Is it visible to others?
(11) Can I wear different types of clothing with the device?
(12) Is it suitable for continuous monitoring 24/7?
(13) Will it remain in place or accidentally detach?
(14) Will it interfere with normal activities of daily life?

### 2.4. Statistical Analysis

Three statistical analyses were carried out. First, preferred placements of IMUs and the preferred garment were identified, using the percentages of each body part/garment that were selected across participants. Second, we calculated the percentage of responses to the 14 questions for each STS and each Likert-scale level. Responses to the 14 questions for the STS were compared between genders using Radar charts (showing mean values relative to each level of Likert-scale levels). These charts were used to facilitate qualitative comparisons between genders, since the small sample size did not support a rigorous statistical assessment. Third, separate paired *t*-tests were performed to compare responses to the 14 questions between the STS and IMU systems [66]. In the latter, a *p*-value < 0.01 was considered significant (a conservative value was used, given the large number of comparisons).

## 3. Results

### 3.1. Preferred Placement and Garment

Table 4 summarizes the results regarding preferred placements of IMUs and the STS, which are shown as percentages for each body part that was selected. For the placement of an IMU, the ankle, wrist, shank, foot, thigh, and waist were the most frequently selected locations. A low percentage of participants selected use of an IMU as an external device “anywhere” on their body. In contrast, a large percentage (44.4%) of participants chose “any garment” on their body for an STS. The most frequently selected garments for an STS were short-sleeved T-shirts, wristbands, socks, sleeveless T-shirts, and ankle sleeves.

### 3.2. Usability Questions

In addition to selecting the preferred placement location and garment, the participants were asked to respond to 14 usability questions in two system-specific questionnaires. Table 5 summarizes the responses to each question. These responses indicated that the major of participants strongly agreed with Q3, Q13, and Q14. However, participants were in less agreement with Q9 and Q10. 

**Figure 2:**
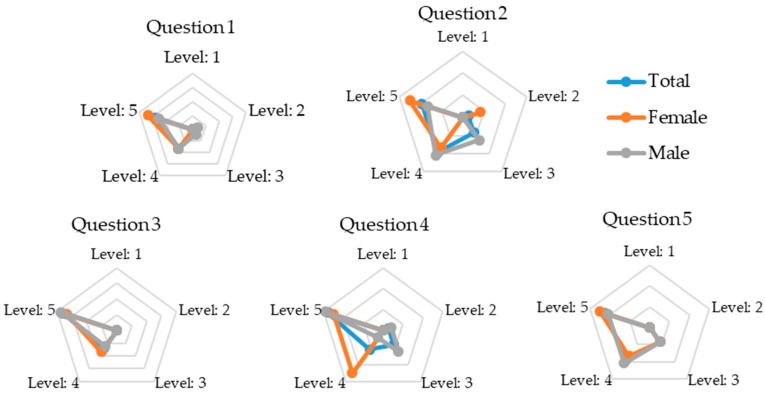

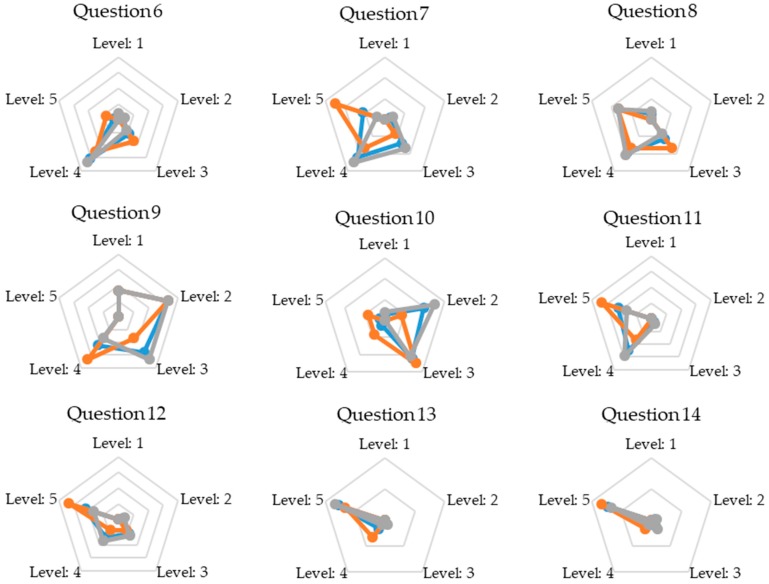
Radar charts showing mean responses to the 14 questions related to the STS for three subject groups: total, female, and male. Levels 1 to 5 refer to the five-level scales used for each question (e.g., 1 = strongly disagree and 5 = strongly agree).

A qualitative comparison between genders for mean responses to the 14 questions for the STS was provided in Figure 2. Responses to questions 1, 3, 5, 6, 8, 11, 13, and 14 were largely similar for both males and females. In contrast, there were discrepancies between genders for the remaining questions, especially Q4, Q7, and Q9. In general, however, responses from each subject group (male, female, and total responses) displayed relatively similar tendencies.

**Figure 3:**
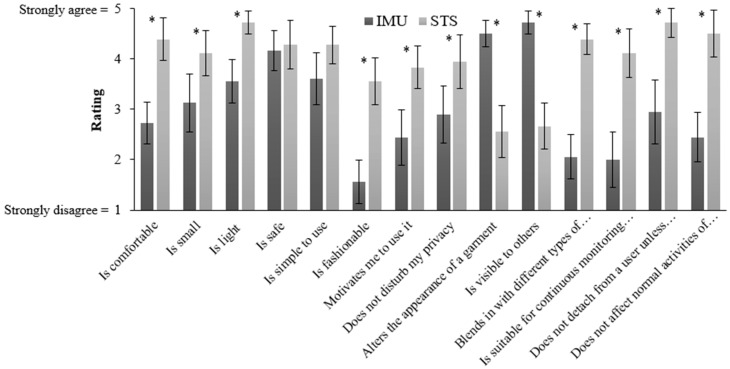
Mean responses to several usability questions regarding an IMU and STS. Note that * indicates a significant difference (*p* < 0.01) in responses between IMUs and STS, and error bars indicate standard deviations.

Figure 3 summarizes responses to the usability questions for IMUs and STS. There were statistically significant differences between mean responses to all questions, with the exceptions of Q4 (“Is it safe”) and Q5 (“Is it simple to use)”. Overall, these responses indicated that participants perceived the STS to be more comfortable, smaller, simpler to use, more fashionable, less visible to others, and more suitable for continuous monitoring. Moreover, the responses suggested that the STS was perceived as a system that would be more likely to be useful for continuous monitoring, and less likely to alter the appearance of a garment. Finally, participants reported that the STS was less likely to negatively impact normal activities of daily life and would be more stable on the body.

## 4. Discussion

Our aim was to evaluate the usability of STSs, which was done via comparisons between two distinct systems, and in comparison with a commercial IMU system. Results were obtained from 18 participants who performed a range of laboratory-based activities, such as sitting, standing, walking, running, performing occupational tasks, and engaging in simple upper-body motions. While completing these activities, they wore two distinct STSs (an SUS and smart socks), as well as a wireless full-body IMU system. After approximately three hours wearing these two systems, participants answered two-part questionnaires to indicate their preferred placement of the IMU system and preferred garments, and to indicate their usability preferences.

Preferred Placement and Garment Choice: Preferred placements for the IMU system and favored STS garment differed among participants; however, a wearable system, such as a garment, was considered superior (Table 4). Interestingly, the short-sleeved shirt was selected as the preferred garment for an STS. To a certain degree, this finding supports that of Bergmann et al. [60], who reported that the most desired locations for wearable sensors were the wrist, arm, torso/abdomen, and waist. However, participants for their questionnaire-based investigation were recruited online and thus may not have had any experience in actually wearing sensors. For accelerometry-based devices, while the hip (waist) is often a top choice because it includes the largest muscles in the body [67,68], patients and users have complained about placement of STS devices in these areas [69]. Furthermore, one report indicated that sensors could not be reliably fixed on the waist during the day because of trunk movements [68]. A second top choice for accelerometry-based devices is the non-dominant wrist [69], despite the fact that this positioning may not be appropriate for wearers using assistive devices [4]. Similarly, the placement of IMUs is also debatable, since the accuracy of physical activity measurements using IMUs differs for various sensor positions [70]. While prior studies have facilitated a greater understanding of the potential for and shortcomings of activity-monitoring systems, none, to the best of our knowledge, have distinguished between intrinsic and extrinsic wearable sensors, and most appear to have focused on the latter. In contrast, the current investigation differentiated between intrinsic and extrinsic wearable devices. 

Usability: The relative usability of intrinsic and extrinsic wearable systems is an important factor in the design and use of wearable systems. To date, such studies have not compared usability between an STS and an IMU systems (as respective examples of intrinsic and extrinsic types), while earlier studies have evaluated the usability of wearable sensors [60,71]. Our study was designed to address this limitation. As summarized in Table 5, participants appeared confident that a wearable STS would not affect their normal activities of daily life and would not detach during the day. Furthermore, the weight of an STS was not a concern for them, and they indicated that they would feel comfortable and safe while using an STS for their activities and healthcare purposes. Moreover, they agreed moderately that the STS was fashionable, simple to use, and suitable for continuous monitoring. However, they were in doubt to some extent as to whether an STS would be visible to others or alter the appearance of a garment. There were also suggestions of several potential gender differences in our findings. As indicated in Figure 2, females and males had slightly different reactions to the STS in terms of usability, though both agreed on the utility of the devices. Gender differences were more recognizable in responses to three questions regarding their views about the safety, motivation, and physical attractiveness of the STS. However, results of our comparison between the STS and IMU systems (Figure 3) suggested a clear and general preference for the STS, with the exception of findings for Q4 and Q5, which indicated that participants agreed that both systems would be safe and simple to use.

Although this study first investigated the usability of STS in comparison to a commercial IMU system, which indicated a relative preference for an STS system, several limitations should be addressed in future studies. First, all 18 participants were young and healthy, and therefore lacked common physical concerns of older-aged adults (arthritis, limited range of mobility, etc.); thus, the results provided herein may not be suitable for older adults. Second, questions related to the appearance of the STS garment (e.g., is it fashionable?) should only be cautiously extended to other age ranges, since these aspects could be correlated with age. Third, the numbers of females and males were not balanced, requiring further analysis to better understand whether usability perspectives differ substantially between genders. Fourth, we collected data in a laboratory setting over a three-hour period; as such, future studies are needed to evaluate these wearable devices over an extended period of time, and in real-life settings and situations. Finally, we used only a single IMU system, which was a commercially available full-body monitoring system. We caution that most available IMU-based devices or activity monitors are designed for a specific body segment. We also used fairly large IMU sensors. Therefore, usability results for the IMU system tested here may not extrapolate to other accelerometry-based or IMU-based devices, and future study may need to evaluate the usability of smaller sensors. 

Despite the fact that a number of STSs have been developed to monitor physiological factors and activities [1,32,35,72], they are by no means exhaustive in terms of the data they are capable of capturing. For example, there is currently no type of STS that can directly measure acceleration and velocity. Nonetheless, it may be possible to address this limitation in the future by integrating accelerometers and IMUs into fabric, either intrinsically (sensing features are applied to fibers or yarns) or extrinsically (utilizing sensing objects that are applied or attached on the textile surface). Consider, for instance, the work of Lorussi et al. [54], who recently developed a wearable device called INTERACTION using IMUs, knitted piezoresistive sensors, and textile Electromyography electrodes. This wearable device is composed of a shirt, a glove, and trousers, and was designed to detect a range of specific common activities (e.g., gait, grasping, balance, upper and lower arms activities, and reaching activities). Furthermore, flexible electronics, such as tattoo devices, are certainly a promising area of research [73,74]. Though this technology is not widely available for commercial products, it has been used for diverse application, such as measuring heart, brain, and muscles electrical activities [75], and monitoring vital signs and electrophysiology parameters [76]. While we did not test such a system here, it has the potential for future application. Thus, future work should be performed to assess the usability of such systems.

In recent years, researchers have been developing diverse STSs and expanding their utility for industrial and healthcare applications. This is evidenced, for example, by the fact that the number of papers with the keywords “e-textile”, “textile sensor”, “smart textile”, “smart garment”, or “smart fabric” (via Scopus, May 2018) was 853 in the last decade (2008–2017), compared to 411 up to 2007. Furthermore, global market estimates related to the development of STSs were $289.5 million in 2012, but are expected to exceed $1.5 billion by 2020 [77]. In short, the market for STSs has exploded in recent years, which is spurring expanded research in activity monitoring systems [29]. Furthermore, ongoing advances in materials, textiles, and miniaturized electronics result in a range of new STSs for use in diverse settings. However, a wearable sensing system will not be optimally effective unless the user feels comfortable wearing it and is confident that it can perform well under the demands of normal use. Our study confirmed that both hardware and usability are factors that must be taken into account for the future development of smart textile systems.

## 5. Conclusions

We assessed the placement and usability of two STSs, including an SUS and commercial Sensoria socks, and we compared them to a commercial whole-body IMU system. Questionnaire responses were obtained from 18 study participants, after three hours of wearing these systems while completing diverse activities, in order to compare and contrast the two systems across a range of usability factors. A majority of participants indicated that they would prefer to wear “any garment” on their body as an STS device in contrast to wearing an IMU system. In particular, the short-sleeved T-shirt, wristband, socks, sleeveless T-shirt, and ankle sleeve were the preferred garments for an STS. Overall, individuals preferred wearing an STS to wearing an IMU system, indicating that the STS was advantageous over the IMU in several aspects of usability.

## 6. Patents

A US patent has been filed for the SUS; Disclosure # 62/641,448.

## Figures and Tables

**Table 1:** Diverse types of MEMS-based wearable sensors developed to measure human kinematics.

Types of Wearable Sensor by MEMS	Studies	Commercial Devices
Accelerometer	Foerster, et al. [19]	ActivPAL (PAL Technologies Ltd, Glasgow, Scotland, UK), ActiGraph (ActiGraph, Pensacola, FL, USA), Actical (Philip, Andover, MA, USA), GENEActive (Activinsights, Kimbolton, Cambridgeshire, UK), Omron (OMRON, Healthcare Europe B.V., Hoofddorp, The Netherlands), StepWatch (Orthocare Innovations, WA, USA)
Gyroscope	Aminian, et al. [20] Sabatini, et al. [21]	InterSense (InterSense Billerica, MA, USA), Sparkfun (SparkFun Electronics, Boulder, CO, USA)
Magnetic sensor	Finley and Lee [22] Ramanathan, et al. [23]	InterSense
Inertial sensor	Luinge [12]	Xsens
Inertial Measurement Unit	Roetenberg, et al. [13] Mokhlespour, et al. [24]	Xsens, Biosyn System, APDM (APDM, Inc., Portland, OR, USA)
Multi-sensing devices	English, et al. [25] Steene-Johannessen, et al. [26]	SenseWear (BodyMedia Inc., Pittsburgh, PA, USA) and Actiheart (CamNtech, Cambridge, United Kingdom)

**Table 2:** Some textile sensors and systems for medical applications.

Project/Products	Description	Year	Ref.
VTAMN	Smart shirt to monitor physiological parameters	2004	[40]
WEALTHY	Smart garment to monitor vital signs (i.e., respiration, and electrocardiogram) and human activity	2005	[41]
MARSIAN	Smart glove to measure temperature	2005	[42]
MyHeart	Two smart garments to monitor activities and respiration	2007	[43]
BIOTEX	Device to monitor physiological factors and body fluids	2008	[44]
ConText	Vest to monitor muscle activity continuously	2008	[45]
OFSETH	Device to monitor chest movements during respiration	2009	[46]
Sensatex	Smart shirt to monitor vital signs (i.e., heart and respiration rates) and movement	2009	[47]
ProeTEX	Smart garment for emergency workers to measure vital health parameters, such as heart rates, respiration rates, and postures	2010	[48]
SenseWear Body Armband (SAB)	Device to measure energy expenditure	2010	[49]
Texisense	Smart sock to measure foot pressure	2015	[50]
Sensoria	Smart sock to measure foot pressure	2015	[51]
Alpha-Fit	Smart sock to measure foot pressure	2015	[52]
Body Worn Sensor	Smart textile sensor developed by electroactive polymers	2016	[53]
INTERACTION	Shirt and trousers for monitoring activities of daily living	2016	[54]
Trunk Motion system (TMS)	Smart shirt to measure 3D angles of trunk movement	2017	[55]
Knitted Glove Sensing System	Smart glove to capture finger movements using compression strain	2018	[56]
Smart Undershirt (SUS)	Smart undershirt for task classification and angle prediction of upper body motion	2018	[57]

**Table 3:** Summary information about study participants.

Item	Mean	Std. Dev.	Range
Age (years)	21.9	3.3	18–30
Body Mass (kg)	76.5	7.6	64.4–86
Stature (cm)	173	6.5	165–186
BMI (kg/m^2^)	25.9	2.6	22.5–29.8

**Table 4:** Preferred placement of IMUs and preferred garment for an STS.

IMU	%	STS	%
Anywhere on my body	5.6	Any garment on my body	44.4
Anywhere on my upper body	0	Any garment on my upper body	11.1
Anywhere on my lower body	5.6	Any garment on my lower body	16.7
Head	38.9	Hat	33.3
Neck	0	Headband	38.9
Shoulders	27.8	Neckband	22.2
Torso/Abdomen	11.1	Sleeveless T-shirt	44.4
Back	33.3	Short-sleeved T-Shirt	61.1
Chest	27.8	Long-sleeve T-shirt	38.9
Waist	44.4	Elbow sleeve	27.8
Upper Arms	27.8	Wristband	55.6
Lower Arms (Forearm)	27.8	Glove	27.8
Elbow	5.6	Finger band	33.3
Wrist	55.6	Underwear	33.3
Hand	27.8	Shorts	38.9
Finger (s)	0	Trousers/pants	33.3
Hip	22.2	Thigh sleeve	33.3
Thigh	44.4	Knee sleeve	38.9
Knee	33.3	Ankle sleeve	44.4
Shank (lower leg)	50	Socks	55.6
Ankle	66.7		
Foot	44.4		

**Table 5:** Percentages for responses to 14 usability questions for the STS.

	Questions	1 Strongly Disagree (%)	2 (%)	3 (%)	4 (%)	5 Strongly Agree (%)
1	Is comfortable	0	6	6	33	55
2	Is small	0	6	16	39	39
3	Is light	0	0	0	28	72
4	Is safe	0	6	16	22	56
5	Is simple to use	0	0	17	39	44
6	Is fashionable	6	6	21	61	6
7	Motivates me to use it	0	6	28	44	22
8	Does not disturb my privacy	6	0	22	39	33
9	Alters the appearance of a garment	17	33	28	22	0
10	Is visible to others	6	38	44	6	6
11	Blends in with different types of clothing that might be worn with the device	0	0	6	50	44
12	Is suitable for continuous monitoring 24/7	0	6	22	28	44
13	Does not detach from a user unless needed	0	0	6	16	78
14	Does not affect normal activities of daily life	0	6	11	11	72
